# Magnetic resonance imaging preprocessing and radiomic features for classification of autosomal dominant polycystic kidney disease genotype

**DOI:** 10.1117/1.JMI.10.6.064503

**Published:** 2023-12-27

**Authors:** Linnea E. Kremer, Arlene B. Chapman, Samuel G. Armato

**Affiliations:** a The University of Chicago, Committee on Medical Physics, Department of Radiology, Chicago, Illinois, United States; b The University of Chicago, Department of Medicine, Chicago, Illinois, United States

**Keywords:** autosomal dominant polycystic kidney disease, radiomics, preprocessing, computer-aided diagnosis, magnetic resonance imaging

## Abstract

**Purpose:**

Our study aims to investigate the impact of preprocessing on magnetic resonance imaging (MRI) radiomic features extracted from the noncystic kidney parenchyma of patients with autosomal dominant polycystic kidney disease (ADPKD) in the task of classifying PKD1 versus PKD2 genotypes, which differ with regard to cyst burden and disease outcome.

**Approach:**

The effect of preprocessing on radiomic features was investigated using a single T2-weighted fat saturated (T2W-FS) MRI scan from PKD1 and PKD2 subjects (29 kidneys in total) from the Consortium for Radiologic Imaging Studies of Polycystic Kidney Disease study. Radiomic feature reproducibility using the intraclass correlation coefficient (ICC) was computed across MRI normalizations (z-score, reference-tissue, and original image), gray-level discretization, and upsampling and downsampling pixel schemes. A second dataset for genotype classification from 136 subjects T2W-FS MRI images previously enrolled in the HALT Progression of Polycystic Kidney Disease study was matched for age, gender, and Mayo imaging classification class. Genotype classification was performed using a logistic regression classifier and radiomic features extracted from (1) the noncystic kidney parenchyma and (2) the entire kidney. The area under the receiver operating characteristic curve (AUC) was used to evaluate the classification performance across preprocessing methods.

**Results:**

Radiomic features extracted from the noncystic kidney parenchyma were sensitive to preprocessing parameters, with varying reproducibility depending on the parameter. The percentage of features with good-to-excellent ICC scores ranged from 14% to 58%. AUC values ranged between 0.47 to 0.68 and 0.56 to 0.73 for the noncystic kidney parenchyma and entire kidney, respectively.

**Conclusions:**

Reproducibility of radiomic features extracted from the noncystic kidney parenchyma was dependent on the preprocessing parameters used, and the effect on genotype classification was sensitive to preprocessing parameters. The results suggest that texture features may be indicative of genotype expression in ADPKD.

## 1 Introduction

Autosomal dominant polycystic kidney disease (ADPKD) is the most common hereditary kidney disorder and is responsible for 10% of patients with end-stage kidney disease under the age of 65.^1^^,^^2^ ADPKD is predominantly due to mutations in two genes, PKD1 (85%) and PKD2 (15%). ADPKD results in gradual enlargement of the kidneys due to cyst growth and enlargement over decades prior to decline in kidney function and kidney failure. Increases in kidney size is associated with loss of kidney function in ADPKD, and height-corrected total kidney volume (htTKV) is an imaging prognostic biomarker approved by the U.S. Food and Drug Administration. Typically, PKD1 patients have greater htTKV than PKD2 patients, and although PKD1 and PKD2 kidneys increase in size at the same rate, PKD1 patients have 40% more detectable kidney cysts.^2^ These findings indicate that the rate of cyst formation may account for the differences in htTKV observed between PKD genotypes. This is consistent with the observation that PKD2 patients typically start dialysis 20 years later and live 10 years longer than PKD1 patients. Understanding the nature of alterations in the noncystic parenchyma in PKD1 and PKD2 patients may help to understand genetic differences in disease progression in ADPKD.

Radiomics transforms medical images into mineable, quantitative data through the calculation of image features that range from simple, first-order signal intensity statistics to more complex spatial relationships of signal intensities, such as gray-level co-occurrence matrix (GLCM) features, that differ in mathematical computational complexity. Radiomics analyses of magnetic resonance imaging (MRI) images to evaluate patients with ADPKD have only recently been explored, and promising studies have shown the additive power of texture in predicting future kidney function decline in ADPKD patients.^3^^–^^5^ Radiomic features could capture textural alterations in the noncystic compartments of the kidney due to differences in cystogenesis or tissue response to injury and add value to established kidney size differences. Although the power of radiomics enhances the understanding of a disease, the field lacks a standardized approach to extracting features. Imaging and radiomics workflows involve image acquisition and reconstruction, segmentation, image processing, feature computation, and statistical modeling.^6^ Image processing is an important part of the radiomics workflow; it harmonize images before feature extraction and includes operations such as image resizing, pixel resampling, gray-level discretization, and filtering, which all have downstream effects on feature values.^7^^,^^8^ Qualitative MRI sequences (e.g., T1-weighted and T2-weighted) require a normalization or signal intensity standardization process for inter- and intrapatient radiomic comparisons due to arbitrary signal intensities. In ADPKD imaging studies, T2-weighted fat saturated (T2W-FS) MR images are used due to their superior cyst-to-parenchyma contrast; however, previous ADPKD radiomic studies differed in their preprocessing, specifically MR normalization and gray-level discretization methods.^3^^–^^5^ Disease-specific radiomic studies, such as brain and prostate cancer, have investigated the effect of preprocessing on radiomic features, but the effect of preprocessing parameters on ADPKD radiomic features is currently unknown.

The purpose of this work is twofold: (1) to assess the impact of preprocessing on radiomic features and (2) to evaluate the ability of features extracted from the noncystic kidney parenchyma to classify PKD1 and PKD2 variants of ADPKD.

## 2 Methods

### 2.1 Databases for Feature Reproducibility and Genotype Classification

This work analyzed images that were obtained from two previously completed prospective imaging studies: the Consortium for Radiologic Imaging Studies of Polycystic Kidney Disease (CRISP) study, a longitudinal study of cyst and kidney growth in a large cohort of patients with ADPKD, and the HALT Progression of Polycystic Kidney Disease (HALT PKD) randomized clinical trial (NCT00283686). T2W-FS MR images were acquired on 1.5T scanners using single-shot fast spin echo/half-Fourier acquisition single-shot turbo spin echo imaging with fat saturation and a 3 mm fixed slice thickness. Representative 2D MR images of the left and right kidney were chosen based on the coronal MR image that maximized the longitudinal length for each kidney individually.

The CRISP study was first used to evaluate the effect of preprocessing on radiomic features. T2W-FS MR images from 15 subjects (7 with the PKD1 genotype and 8 with the PKD2 genotype) were analyzed. The images were acquired between 2001 and 2002 from a single clinical site and were matched for age, sex, and htTKV. Image matrix sizes for all CRISP images were 256×256  pixels, with pixel sizes ranging from 1.17 to 1.37 mm. Table 1 shows the subject characteristics.

**Table 1:** CRISP subject characteristics.

Clinical	PKD1	PKD2
No. patients	7	8
Mean age ± SD		
Male (n=7)	23±7.79	27±6.80
Female (n=8)	25±9.42	24±7.76
MIC mean htTKV ± SD		
1A (n=0)	—	—
1B (n=8)	358.12±55.94	283.28±67.45
1C (n=4)	274.71±2.34	358.23±74.86
1D (n=1)	320.09	—
1E (n=2)	451.74	428.29

The HALTA-PKD randomized clinical trial was then subsequently used for genotype classification. A dataset of 136 age-, gender-, and Mayo imaging classification (MIC)-matched baseline MR images were analyzed.^9^ Due to the low prevalence of PKD2 to PKD1, the HALTA-PKD dataset had 68 PKD2 patients who had measured htTKV, baseline MR images, and the ability to match with PKD1 patients for age, gender, and MIC. The MR images had been acquired from seven different sites between 2006 and 2009. All HALT MR image matrix sizes ranged from 256×256 to 560×560  pixels, and pixel sizes ranged from 0.63 to 1.8 mm. Table 2 shows the subject characteristics.

**Table 2:** HALT subject characteristics for classification. A p<0.05 was significant using the Wilcoxon rank sum test.

Clinical	PKD1	PKD2	p-value
No. patients	68	68	
Mean age ± SD			
Male (n=64)	40±7.40	41±6.86	0.81
Female (n=72)	40±7.85	41±7.77	0.61
MIC mean htTKV ± SD			
1A (n=10)	257.61±41.28	231.38±37.34	0.31
1B (n=60)	408.66±90.41	380.98±91.28	0.28
1C (n=48)	651.07±198.56	681.08±247.64	0.78
1D (n=14)	1075.01±258.18	1064.88±287.25	0.90
1E (n=4)	1453.34±22.68	1627.36±349.44	1.0

### 2.2 MR Image Preprocessing

For qualitative MRI, normalization is used to harmonize images that come from different sites and scanners that may result in large variations in signal intensities. The image normalization methods chosen were (1) the z-score method and (2) the reference-tissue method. Z-score normalization uses the mean and standard deviation of the entire image to normalize signal intensities:

$$X_{z\text{-}\text{score}}=\frac{X_{\mathrm{gl},i}-\mu }{\sigma },$$ (1)

where the μ represents the mean of the entire image, σ represents the standard deviation of the entire image, $X_{\mathrm{gl},i}$ represents the pixel of interest, and $X_{z\text{-}\text{score}}$ represents the normalized gray level of the pixel intensity. Reference-tissue normalization uses a healthy tissue to standardize the gray levels in an image. The chosen reference-tissue method transformed a region of interest (ROI) mean and standard deviation extracted from the psoas muscle in each image to have a mean of 100 and a standard deviation of 10. Muscle is hypointense on T2-weighted MR images and has been used in previous studies to standardize MR signal intensity.^10^^–^^12^ The original images were also used for feature extraction without any normalization applied.

All images were resized to 256×256 for feature extraction using nearest-neighbor interpolation. Additionally, pixel sizes were harmonized by upsampling to 1.0×1.0  mm and downsampling to 2.0×2.0  mm using nearest-neighbor interpolation. According to the imaging biomarker standardization initiative (IBSI), it is not known whether upsampling or downsampling schemes are preferable.^6^

3D Slicer, an open-source image-analysis software package, was used for the segmentation of the kidney and cysts, respectively.^13^ Kidneys were segmented manually, and cysts were semiautomatically segmented and removed from the resultant kidneys segmentation to obtain pixel intensities from the noncystic kidney parenchyma.

### 2.3 Feature Extraction

**Fig. 1:**
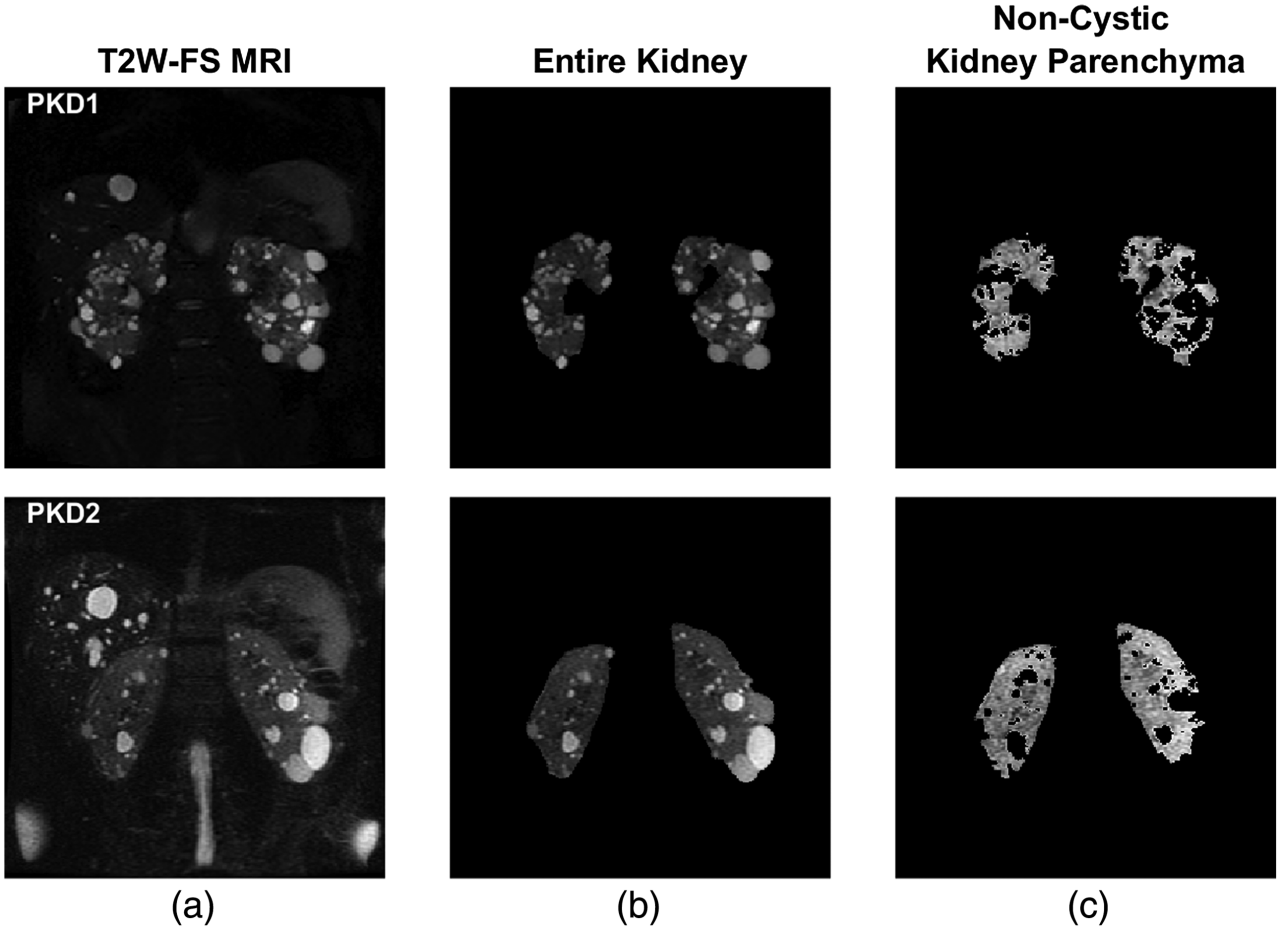
(a) Representative MRI slice, (b) the result of kidney segmentation, and (c) the result of cyst segmentation. These segmented regions were used for feature extraction for an MIC 1B PKD1 patient (top) and a PKD2 patient (bottom).

Pyradiomics was used for feature extraction of the images.^14^ Features were extracted from the noncystic kidney parenchyma and the entire kidney parenchyma (Fig. 1). Ninety-three features were extracted per kidney: first-order (18), GLCM (24), gray-level run length matrix (GLRLM) (16), gray-level size zone matrix (GLSZM) (16), neighboring gray-tone difference matrix (NGTDM) (5), and gray-level dependence matrix (GLDM) (14). The discretization method used in this work was fixed bin size (FBS) as implemented in Pyradiomics:^14^

$$X_{\mathrm{fbs},i}=\lfloor \frac{X_{\mathrm{gl},i}}{W}\rfloor -\lfloor \frac{\min(X_{\mathrm{gl}})}{W}\rfloor +1,$$ (2)

where $X_{\mathrm{gl},i}$ and $X_{\mathrm{fbs},i}$ are the gray level of voxel i before and after discretization, respectively, with bins equally spaced from 0 and $\lfloor \frac{\min(X_{\mathrm{gl}})}{W}\rfloor +1$ to ensure that the minimum gray level for feature extraction is always 1 after discretization. To analyze the impact of normalization on radiomic features from images with different signal intensities, bin widths (W) were calculated using the mean signal intensity range of patient T2W-FS MR images:

$$W=\frac{{\text{range}}_{\text{mean}}}{N_{b}},$$ (3)

where the ${\text{range}}_{\text{mean}}$ is the mean gray-level range across all patient MR images and $N_{b}$ is the number of gray levels or bins for feature calculation. In this work, six gray level bins were analyzed for the noncystic kidney parenchyma and entire kidney: 8, 16, 32, 64, 128, and 256. A difference between pyradiomics implementation of FBS and IBSI is that pyradiomics ensures that the miniumum gray level starts at 1, whereas IBSI starts at the minimum gray-level intensity in the ROI.^6^^,^^14^ Gray-level discretization is used to suppress noise while retaining important biological variation in the ROI and making the feature calculation time more efficient.^6^ An accepted range in the literature for the total number of bins for discretization is between 8 and 128, and this range is frequently used in studies that have previously investigated the effect of gray-level discretization on MRI radiomic feature repeatibility and reproducibility in other disease cohorts.^15^^–^^17^

### 2.4 Feature Reproducibility

To evaluate the reproducibility of features across normalization and gray levels for upsampling and downsampling schemes, the intraclass correlation coefficient (ICC) was calculated for each radiomic feature extracted from the images of the CRISP dataset. The ICC metric has been used in previous radiomics literature to evaluate radiomic feature reproducibility based on test–retest, intrarater, and interrater analyses.^16^^,^^18^^,^^19^ This statistical metric combines information about the degree of correlation and the agreement between measurements. A two-way mixed effects, consistency, single-rater model was used:

$$\mathrm{ICC}=\frac{{\mathrm{MS}}_{R}-{\mathrm{MS}}_{E}}{{\mathrm{MS}}_{R}+(k-1){\mathrm{MS}}_{E}},$$ (4)

where ${\mathrm{MS}}_{R}$ is the mean square for observations, ${\mathrm{MS}}_{E}$ is the mean square for error, and k represents the “raters of interest,” which are the MR normalization methods used (z-score, psoas, and the original image). ICC can take values between 0 and 1, with values closer to 1 representing stronger reproducibility; currently, there is no standard ICC value for “acceptable” reproducibility of radiomic features, and this determination differs across the radiomics literature. ICC values were calculated in MATLAB, and according to Koo and Li,^20^ values <0.5 indicate poor reproducibility, 0.5 to 0.75 indicate moderate reproducibility, 0.75 to 0.9 indicate good reproducibility, and >0.9 indicate excellent reproducibility.^21^ In this work, good and excellent reproducibilities were combined, so ICC values in the range [0.75 to 1.0] were classified as good-to-excellent reproducibility.

### 2.5 Feature Selection and Classification

A logistic-regression classifier using fivefold cross validation was utilized on the HALT dataset only to classify genotype. For each iteration, the top-10 performing radiomic features of the training partition were determined using the area under the receiver operating characteristic curve (AUC). A Pearson correlation coefficient threshold of 0.7 was used to remove correlated features during feature selection. This process used a repeated cross-validation (rCV) of 10 to account for the variance in k-fold cross-validation.

## 3 Results

### 3.1 Reproducibility of Radiomic Features Across Preprocessing

**Fig. 2:**
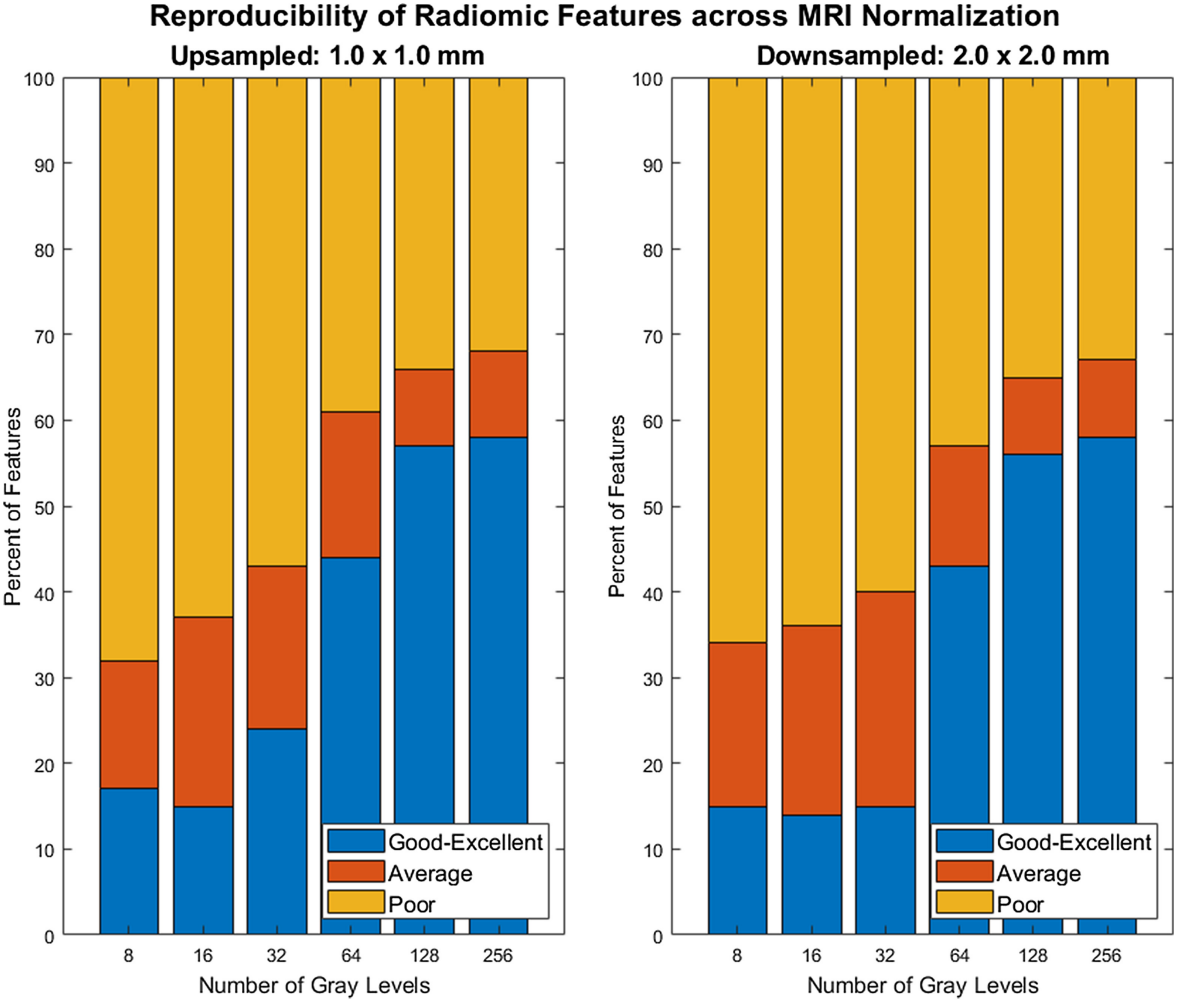
ICC scores for radiomic features (poor, moderate, and good-to-excellent) across MRI normalizations.

Figure 2 shows the variability of ICC scores of CRISP-derived radiomic features across normalizations and gray-level discretization using FBS discretization for both upsampling and downsampling schemes. As the bin counts increase (smaller bin widths and an increase in gray levels for feature computation), feature reproducibility across normalizations also increases. Upsampling and downsampling methods under 64 bins yielded poor reproducibility for over 50% of the total features calculated. For both resampling methods, the largest increase in the number of features with good-to-excellent reproducibility was over 20% from 32 gray levels to 64 gray levels. Table 3 shows the mean ICC values across feature families; an increase in reproducibility is shown with increasing gray levels, the highest being features calculated with 256 gray levels. Increasing the gray levels of the ROI resulted in larger mean ICC values across feature families except for first-order features.

**Table 3:** Mean ICC scores for radiomic features across feature families using upsampling and downsampling methods.

Number of gray levels	8		16		32		64		128		256	
	1.0	2.0	1.0	2.0	1.0	2.0	1.0	2.0	1.0	2.0	1.0	2.0
First order	0.33	0.33	0.33	0.33	0.33	0.33	0.36	0.36	0.38	0.38	0.38	0.38
GLCM	0.60	0.61	0.62	0.61	0.62	0.60	0.65	0.63	0.72	0.72	0.77	0.76
GLDM	0.55	0.59	0.58	0.59	0.65	0.64	0.76	0.75	0.84	0.84	0.86	0.85
GLRLM	0.58	0.60	0.56	0.56	0.63	0.60	0.78	0.75	0.85	0.84	0.87	0.86
GLSZM	0.56	0.56	0.50	0.58	0.66	0.60	0.77	0.74	0.85	0.85	0.87	0.87
NGTDM	0.54	0.58	0.56	0.58	0.55	0.54	0.60	0.62	0.76	0.77	0.80	0.81

There were seven features that exhibited good-to-excellent (ICC>0.75) reproducibility across all gray levels for both upsampling and downsampling: first-order skewness and kurtosis, GLCM inverse difference (ID) matrix normalized and ID normalized, GLDM dependence nonuniformity, GLSZM gray-level nonuniformity, and NGTDM coarseness. Two features, GLCM maximum correlation coefficient (MCC) and GLSZM small area emphasis, were reproducible across all gray levels for upsampling only.

**Fig. 3:**
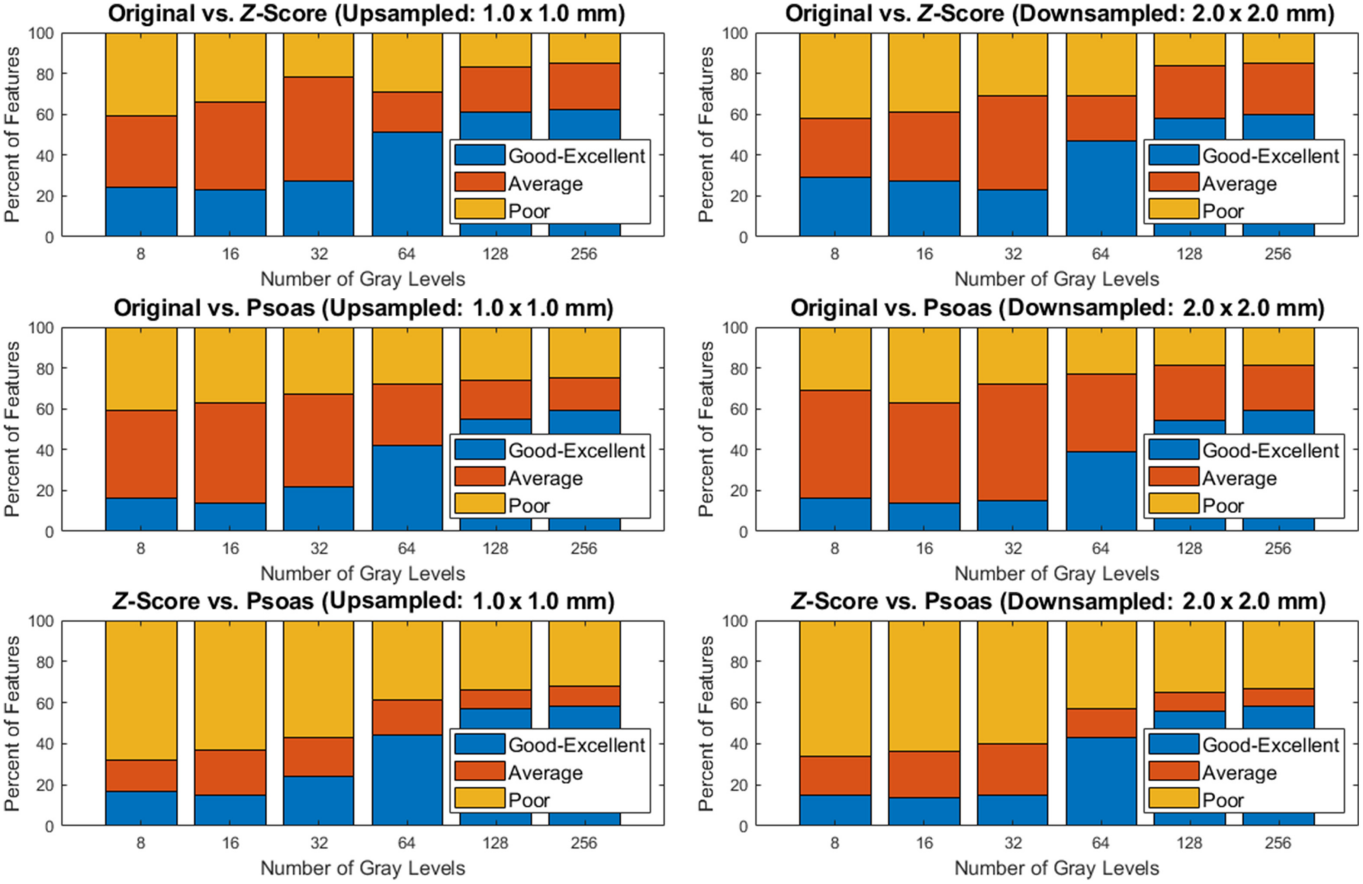
Percentage of total radiomic features for pairwise comparisons of MRI normalizations that were poor, moderate, and good-to-excellent.

Figure 3 contains the ICC values that were observed using pairwise comparisons of MRI normalization. ICC values of radiomic features using z-score normalization and psoas normalization had a larger percentage of features with poor reproducibility, ranging from 32% to 68%, versus original and z-score of 15% to 42% and the original image and psoas normalization of 19% to 41%. A similar trend for all pairwise comparisons in Table 3 was that increasing the number of gray levels increased the percentage of features with good-to-excellent reproducibility across normalization pairwise comparisons.

### 3.2 Noncystic Kidney Parenchyma Texture Features for Genotype Classification

**Fig. 4:**
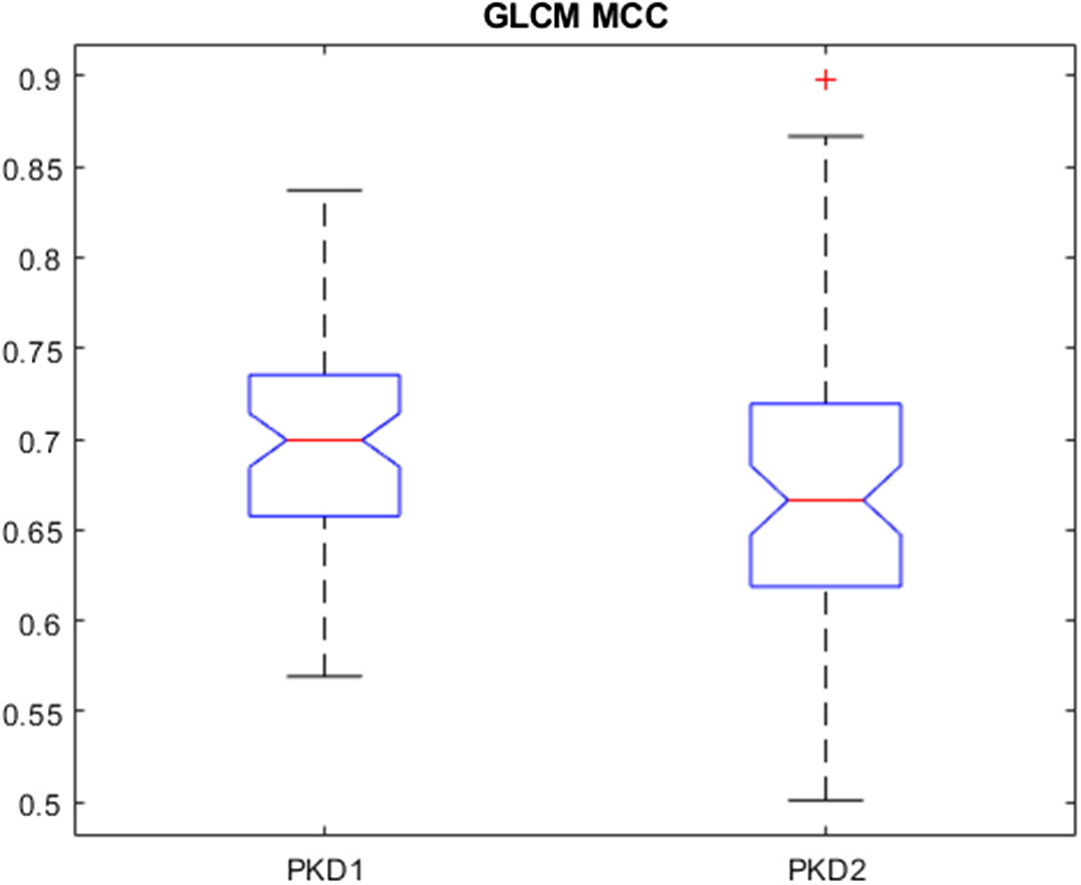
Boxplot of a feature that was frequently used across rCV for z-score normalization with 64 gray levels and downsampling of $2.0\times 2.0\text{-}{\mathrm{mm}}^{2}$. GLCM MCC is a measure of complexity of texture between 0≤MCC≤1.^14^

**Fig. 5:**
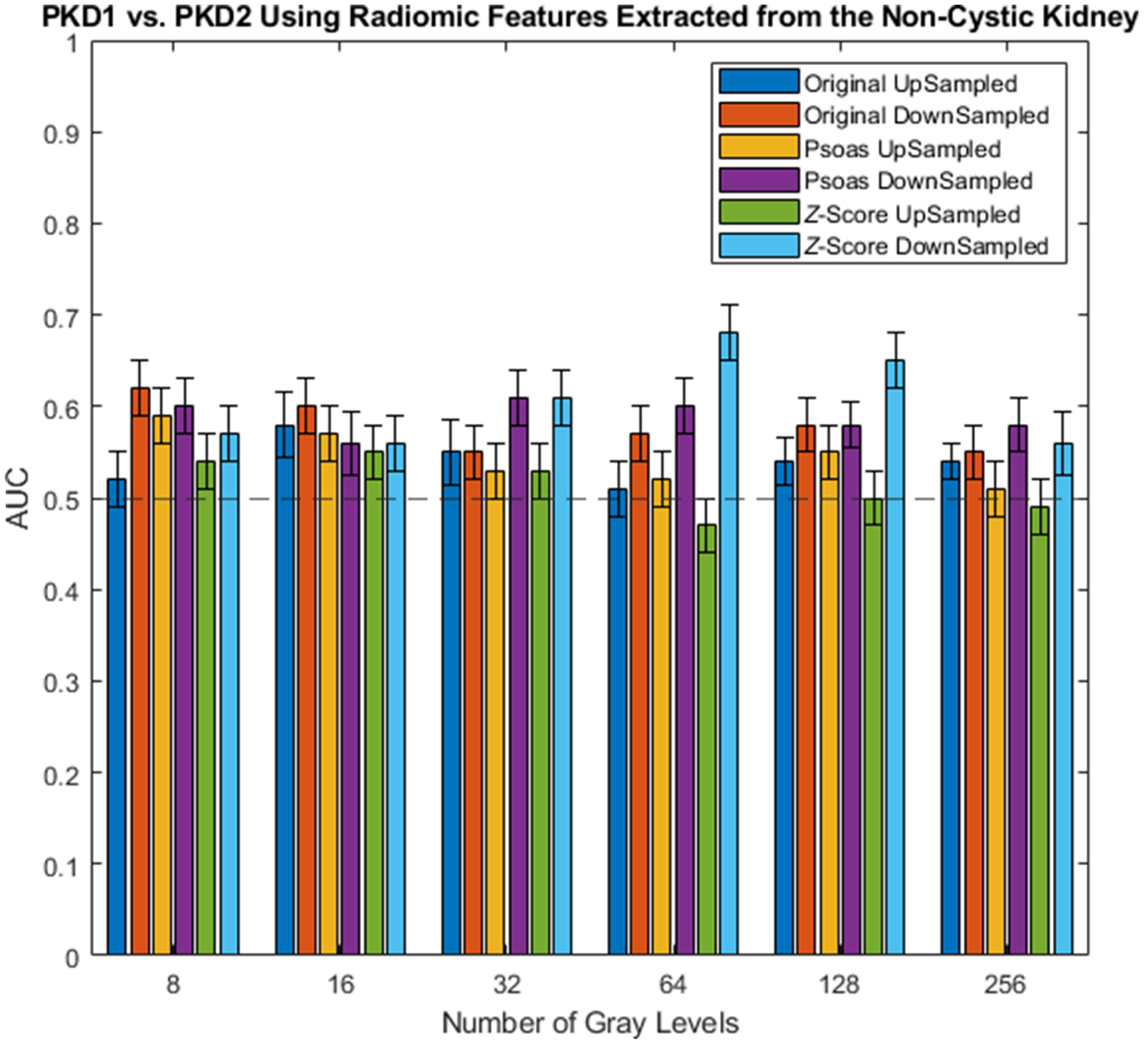
AUC values in classifying genotype using radiomic features extracted from the noncystic kidney parenchyma. The dotted line at an AUC of 0.5 is random guessing.

Tables 4 and 5 show the AUC values across preprocessing parameters using the original image, z-score normalization, and psoas normalization using features extracted from the noncystic kidney parenchyma (Figs. 4 and 5). The range of AUC values was between 0.47 and 0.68. Across normalizations, the range of AUC values for the original image, z-score normalization, and psoas normalization were 0.51 to 0.62, 0.47 to 0.68, and 0.51 to 0.61, respectively. Across normalizations and upsampling only, the ranges of AUC values for the original image, z-score normalization, and psoas normalization were 0.51 to 0.58, 0.47 to 0.55, and 0.51 to 0.59, respectively; the ranges of AUC values for the downsampling method for the original image, z-score normalization, and psoas normalization were 0.55 to 0.62, 0.56 to 0.68, and 0.56 to 0.61, respectively.

**Table 4:** AUC values for upsampling for features extracted from the noncystic kidney parenchyma.

Number of gray levels						
Image	8	16	32	64	128	256
Original	0.52	0.58	0.55	0.51	0.54	0.54
	[0.49, 0.55]	[0.54, 0.61]	[0.51, 0.58]	[0.48, 0.54]	[0.51, 0.56]	[0.51, 0.55]
Psoas	0.59	0.57	0.53	0.52	0.55	0.51
	[0.56, 0.62]	[0.54, 0.60]	[0.50, 0.56]	[0.49, 0.55]	[0.52, 0.58]	[0.48, 0.54]
Z-score	0.54	0.55	0.53	0.47	0.50	0.49
	[0.51, 0.57]	[0.52, 0.58]	[0.50, 0.56]	[0.44, 0.50]	[0.47, 0.53]	[0.46, 0.52]

**Table 5:** AUC values for downsampling for features extracted from the noncystic kidney parenchyma.

Number of gray levels						
Image	8	16	32	64	128	256
Original	0.62	0.60	0.55	0.57	0.58	0.55
	[0.59, 0.65]	[0.57, 0.63]	[0.52, 0.58]	[0.54, 0.60]	[0.55, 0.61]	[0.52, 0.58]
Psoas	0.60	0.56	0.61	0.60	0.58	0.58
	[0.57, 0.63]	[0.52, 0.59]	[0.58, 0.64]	[0.57, 0.63]	[0.56, 0.61]	[0.55, 0.61]
Z-score	0.57	0.56	0.61	0.68	0.65	0.56
	[0.54, 0.60]	[0.53, 0.59]	[0.58, 0.64]	[0.65, 0.71]	[0.62, 0.68]	[0.53, 0.60]

### 3.3 Entire Kidney Parenchyma Texture Features for Genotype Classification

**Fig. 6:**
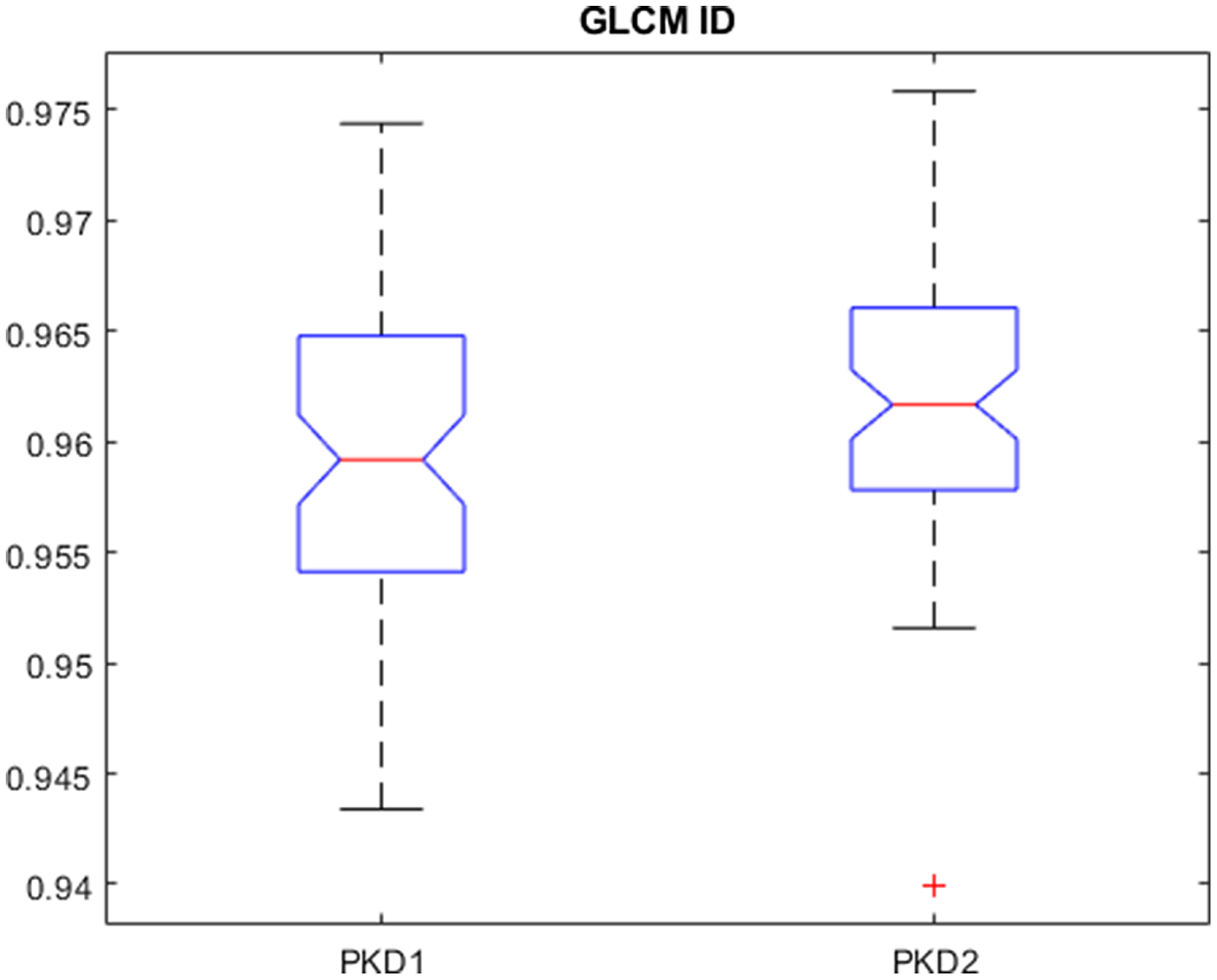
Boxplot of a feature that was frequently used across rCV for psoas normalization with eight gray levels and upsampling of $1.0\times 1.0\text{-}{\mathrm{mm}}^{2}$. GLCM ID is a measure of local homogeneity of the ROI, with a higher value indicating more uniform gray levels.^14^

**Fig. 7:**
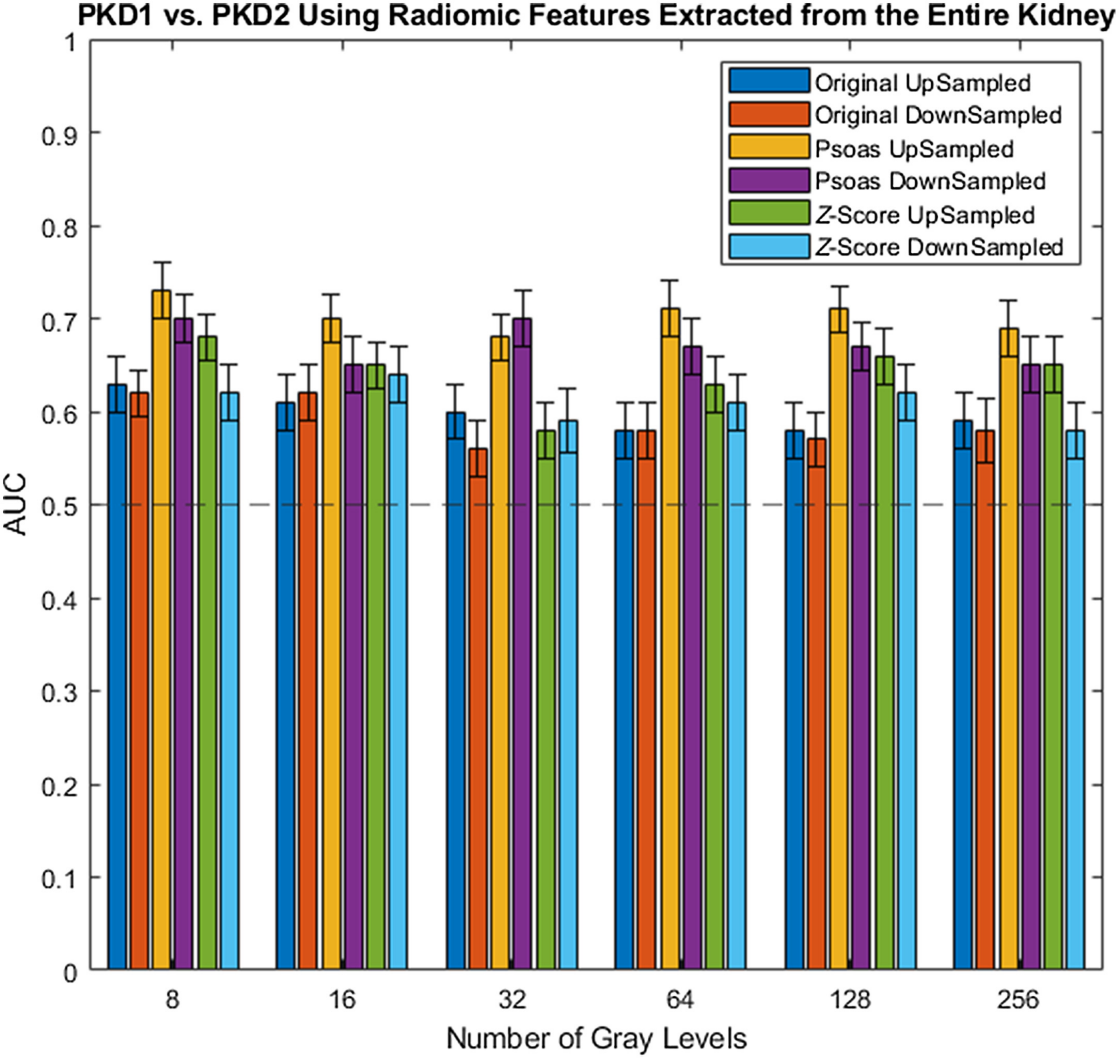
AUC values in classifying genotype using radiomic features extracted from the entire kidney. The dotted line at an AUC of 0.5 is random guessing.

Tables 6 and 7 shows the AUC values across preprocessing parameters using the original image, z-score normalization, and psoas normalization using features extracted from the entire kidney parenchyma (Figs. 6 and 7). The range of AUC values was between 0.56 and 0.73. Across normalizations, the range of AUC values for the original image, z-score normalization, and psoas normalization were 0.56 to 0.63, 0.58 to 0.68, and 0.65 to 0.73, respectively. Across normalizations and upsampling pixel sizes, the range of AUC values for the original image, z-score normalization, and psoas normalization were 0.58 to 0.63, 0.58 to 0.68, and 0.68 to 0.73, respectively; the range of AUC values for the downsampling method for the original image, z-score normalization, and psoas normalization were 0.56 to 0.62, 0.58 to 0.64, and 0.65 to 0.70, respectively.

**Table 6:** AUC values for upsampling for features extracted from the entire kidney parenchyma.

Number of gray levels						
Image	8	16	32	64	128	256
Original	0.63	0.61	0.60	0.58	0.58	0.59
	[0.60, 0.66]	[0.58, 0.64]	[0.57, 0.63]	[0.55, 0.61]	[0.55, 0.61]	[0.56, 0.62]
Psoas	0.73	0.70	0.68	0.71	0.71	0.69
	[0.70, 0.76]	[0.67, 0.72]	[0.65, 0.70]	[0.68, 0.74]	[0.68, 0.73]	[0.66, 0.72]
Z-score	0.68	0.65	0.58	0.63	0.66	0.65
	[0.65, 0.70]	[0.62, 0.67]	[0.55, 0.61]	[0.60, 0.66]	[0.63, 0.69]	[0.62, 0.68]

**Table 7:** AUC values for downsampling for features extracted from the entire kidney parenchyma.

Number of gray levels						
Image	8	16	32	64	128	256
Original	0.62	0.62	0.56	0.58	0.57	0.58
	[0.59, 0.64]	[0.59, 0.65]	[0.53, 0.59]	[0.55, 0.61]	[0.54, 0.60]	[0.54, 0.61]
Psoas	0.70	0.65	0.70	0.67	0.67	0.65
	[0.68, 0.73]	[0.62, 0.68]	[0.67, 0.73]	[0.64, 0.70]	[0.64, 0.69]	[0.62, 0.68]
Z-score	0.62	0.64	0.59	0.61	0.62	0.58
	[0.59, 0.65]	[0.61, 0.67]	[0.56, 0.63]	[0.58, 0.64]	[0.59, 0.65]	[0.55, 0.61]

## 4 Discussion

This work examined preprocessing of MR images and its effect on the reproducibility of radiomic features extracted from the noncystic kidney parenchyma from patients with ADPKD. The ability of these radiomic features to classify patients with PKD1 or PKD2 genetic mutations was explored using both the noncystic kidney parenchyma and the entire kidney. The percentage of radiomic features extracted with good-to-excellent feature reproducibility ranged from 17% to 58% and 15% to 58% across the number of gray levels used for feature calculation for upsampling and downsampling, respectively. In examining the classification performance of patients with either the PKD1 or PKD2 genetic variant across MR normalizations and preprocessing parameters, the AUC values ranged between 0.47 to 0.68 and 0.56 to 0.73 for the noncystic kidney parenchyma and entire kidney, respectively.

To the best of our knowledge, this study is the first to examine the noncystic kidney parenchyma in MR images of ADPKD patients. Xie et al.^22^ recently used radiomic features extracted from the renal parenchyma volume (RPV) on computed tomography to predict kidney function decline. The results of this study showed improved predictive power using texture features rather than TKV or RPV alone, suggesting that noncystic compartments may be informative of future kidney function decline in ADPKD.^22^ Two image normalization methods, z-score normalization and reference-tissue normalization using the psoas muscle, and the original image were chosen for feature extraction across different gray-level discretization and pixel resampling schemes. In the MRI ADPKD radiomics literature, preprocessing parameters are either heterogeneous or not defined, and the effect of preprocessing parameters on radiomic features extracted from the noncystic kidney parenchyma has not been appreciated.

There was an overall trend of increasing mean ICC scores for first-order, GLCM, GLDM, GLRLM, GLSZM, and NGTDM features and the percentage of total features with good-to-excellent feature reproducibility when the number of gray levels was increased in the ROI. In decreasing the number of gray levels for feature extraction, the impact of normalization on the inherent pixel information was evident, showing that the chosen normalization method dramatically affects the original pixel intensity information and downstream feature calculation for all feature families. A pairwise comparison of z-score and psoas normalization obtained poor radiomic feature reproducibility for more than 50% of the radiomic features using 32 gray levels and under; the lowest percentage of radiomic features with poor reproducibility obtained was 32% when discretizing the noncystic kidney parenchyma gray levels to 256. Carré et al.^16^ investigated the impact of the MR normalization method and gray-level discretization on radiomic feature stability from postcontrast 3D axial T1-weighted and axial T2-weighted fluid attenuation inversion recovery images 1 month apart and found that a higher number of bins was associated with a higher number of robust features for both sequences. Among the 93 features extracted across gray levels and resampling schemes, there were 7 features that exhibited good-to-excellent feature ICC scores; the upsampling scheme resulted in 2 additional features, for a total of 9 features that exhibited good-to-excellent reproducibility. Among these features, GLSZM gray-level nonuniformity and NGTDM coarseness have been identified in previous radiomics studies as being correlated with ROI size or voxel number.^12^^,^^23^ The effect of preprocessing on radiomic features from qualitatitive MR images has been investigated in the application of brain and prostate diseases.^12^^,^^16^^,^^17^^,^^24^^,^^25^ Conclusions from these studies differ with respect to optimal preprocessing parameters, showing the unique parameters for a given task, body site, and MR sequence. For example, in the task of brain cancer assessment, radiomics studies have used z-score normalization of MR images and discretizing to 32 gray levels when using FBS discretization and first- and second-order radiomic features, whereas prostate cancer radiomics literature has used between 36 and 42 gray levels with FBS discretization and z-score normalization, histogram matching, or reference-tissue normalization.^16^^,^^25^ Our results show that radiomics features extracted from the noncystic kidney parenchyma using different MR normalization and gray levels for discretization have an effect on radiomic feature reproducibility. These results add to the literature in other disease cohorts investigating the effect of MR preprocessing on radiomic features.

**Fig. 8:**
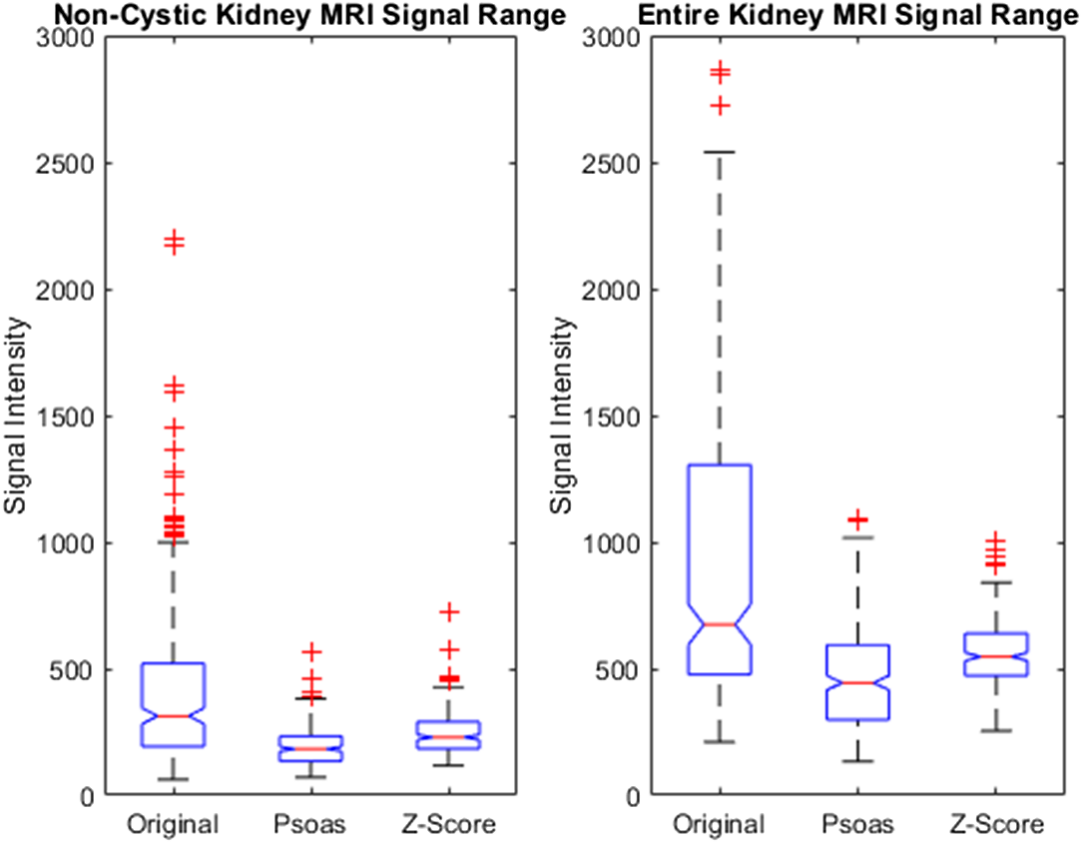
MRI signal intensity range across HALTA-PKD images for all patient data and normalization methods after downsampling. Images obtained were from seven different sites. The noncystic kidney parenchyma has a lower range of signal intensity than the entire kidney parenchyma due to the exclusion of cysts.

Extending these preprocesing parameters to a multisite dataset for the clinical task of genotype classification, the highest AUC values across the number of gray levels for discretization were 0.68, 0.61, and 0.62 for z-score normalization, psoas normalization, and the original image, respectively, for features extracted from the noncystic kidney parenchyma. In addition to the noncystic kidney parenchyma, the entire kidney was also used for feature extraction. Among the preprocessing parameters, the highest AUC values were 0.68, 0.73, and 0.63 for z-score normalization, psoas normalization, and the original image, respectively, for features extracted from the entire kidney parenchyma. Figure 8 shows the effect of normalization on the signal range across patients. The noncystic kidney parenchyma has an overall lower range of signal intensities compared with the entire kidney including the cysts, which tend to be of higher signal intensity and, therefore, with a larger range of values for discretization. The normalization of the MR images tended to improve AUC values using noncystic kidney parenchyma and entire kidney radiomic features for classification. Z-score normalization uses the mean and standard deviation of the entire image and includes signal intensities from cysts, whereas psoas normalization uses the mean and standard deviation of the psoas-muscle that is hypointense in T2W-FS images. Z-score normalization using the downsampling method and 64 gray levels for discretization resulted in the highest AUC value for classification using features from the noncystic kidney parenchyma. When including textural information from cysts, the psoas normalization resulted in the highest AUC values across gray levels and pixel resampling methods. There was not one preprocessing method that optimized the classification performance using the noncystic kidney parenchyma and the entire kidney.

Our study has a few limitations. With respect to preprocessing, there are additional normalization methods, such as histogram-matching and gray-level discrestization methods, namely fixed bin number (FBN), also used in the literature. This work utilized an FBS approach, but currently there is no consensus on the best approach to discretizing gray levels of MR images that have arbitrary signal intensities. For example, IBSI^6^ suggests using the FBN method, but pyradiomics^14^ suggests normalizing MR images and using an FBS approach. FBN is a relative discretizaton approach that normalizes gray levels to the maximum and minimum of the ROI, and using this method on qualitative MR images to extract second-order texture features without any normalization has been implemented in previous studies.^16^ The repeatability and reproducibility of radiomic features using FBN and FBS methods have been investigated in other disease cohorts, with FBS producing more reproducible features.^17^ The results from this study raise an important trade-off in radiomics research of optimal preprocessing parameters for a given task and the effect on feature reproducibility across preprocessing parameters as optimizing the preprocessing parameters to obtain the highest feature reproducibility does not necessarily lead to the best classification.

With respect to the patient data investigated, the impact of mutation type either in functional domains or in mutation strength was not taken into account in this analysis when matching for age, gender, and MIC class. PKD1 patient outcomes differ for truncating and nontruncating mutations, and this variability may impact genotype classification.^26^ With respect to patient data, the number of available patients using the HALT dataset was limited as the prevalence of PKD2 is lower than that of PKD1. The number of PKD2 subjects available to study was small, and a larger sample size would potentially confirm texture differences with greater precision. Adjusting the groups for htTKV provided a very conservative assessment of differences in noncystic parenchyma. However, the purpose here was to evaluate regions of the kidney without the influence of differences in cyst burden. This study provides preliminary information about the potential impact of finding textural changes that associate with genotype in ADPKD.

Future work will include incorporating more image slices as this study included one large, representative, coronal slice of the kidney for analysis. Removing the cysts from the kidney is a tedious process and would benefit from an automated approach. Recent work has utilized semantic instance segmentation to segment kidneys and cysts within the kidney, which may prove useful in providing a fast calculation of the entire kidney and noncystic kidney parenchyma texture.^27^ Utilizing multislice MR texture data of ADPKD kidneys may provide more information of kidney texture rather than a single imaging slice. Additionally, other MR imaging sequences, such as T1-weighted, diffusion-weighted imaging, and quantitative maps could be used to analyze textural differences in ADPKD genotype with respect to mutation type and strength. Retrospective ADPKD MR datasets provide a wealth of imaging data to explore the use of radiomic features and provide valuable insight into the prediction of future kidney function decline, texture differences in genetic mutation, and assessing the longitudinal change in texture over time. However, this work emphasizes the need for ADPKD radiomics studies to state the preprocessing parameters used for feature extraction as they may have a dramatic effect on downstream classification.

Furthermore, this study had a specific clinical task of classifying PKD1 and PKD2 genotypes using radiomic features. However, other clinical tasks in ADPKD could be investigated. For example, texture-based differences in risk-stratified MIC groups in ADPKD, in both the noncystic kidney parenchyma and entire kidney, and optimal preprocessing parameters for feature extraction were investigated, with results suggesting texture-based differences among the risk-stratified MIC classes that warrant further investigation for texture features indicative of faster progressing disease.^28^ Future work will investigate multiclass classification of MIC and differences in genotypes across MIC classes.

## 5 Conclusion

This study investigated the impact of preprocessing on radiomic features extracted from the noncystic kidney parenchyma on T2W-FS MR images. Radiomic features extracted from the noncystic kidney parenchyma were sensitive to MRI normalization, and the results showed that feature reproducibility across MRI normalization is dependent on the number of gray levels available for discretization. Classification performance in distinguishing PKD1 and PKD2 varied with respect to the preprocessing parameters. This work revealed that there are texture features indicative of genotype expression in ADPKD in both the noncystic and entire kidney parenchyma regions. Additionally, there were preferred preprocessing parameters when using features extracted from the noncystic kidney parenchyma compared with those from the entire kidney, and there was not one method that optimized features extracted from either region. The results of this study show the importance of stating preprocessing parameters used for feature extraction as these affect the downstream classification of ADPKD genotype.

## Data Availability

The data used in this study are publicly available upon request through NIDDK Central Repository at https://doi.org/10.58020/956q-m463 and https://doi.org/10.58020/nm6n-qe65. Code is available upon request to lekremer@uchicago.edu.
